# Single-Cell RNA Sequencing Analysis Reveals Metabolic Changes in Epithelial Glycosphingolipids and Establishes a Prognostic Risk Model for Pancreatic Cancer

**DOI:** 10.3390/diagnostics14111094

**Published:** 2024-05-24

**Authors:** Qinwen Ba, Xiong Wang, Hui Hu, Yanjun Lu

**Affiliations:** Department of Laboratory Medicine, Tongji Hospital, Tongji Medical College, Huazhong University of Science and Technology, Wuhan 430074, China

**Keywords:** pancreatic cancer, glycosphingolipids, risk model, single-cell RNA sequencing

## Abstract

Objective: Metabolic reprogramming serves as a distinctive feature of cancer, impacting proliferation and metastasis, with aberrant glycosphingolipid expression playing a crucial role in malignancy. Nevertheless, limited research has investigated the connection between glycosphingolipid metabolism and pancreatic cancer. Methods: This study utilized a single-cell sequencing dataset to analyze the cell composition in pancreatic cancer tissues and quantified single-cell metabolism using a newly developed computational pipeline called scMetabolism. A gene signature developed from the differential expressed genes (DEGs), related to epithelial cell glycosphingolipid metabolism, was established to forecast patient survival, immune response, mutation status, and reaction to chemotherapy with pancreatic adenocarcinoma (PAAD). Results: The single-cell sequencing analysis revealed a significant increase in epithelial cell proportions in PAAD, with high glycosphingolipid metabolism occurring in the cancerous tissue. A six-gene signature prognostic model based on abnormal epithelial glycosphingolipid metabolism was created and confirmed using publicly available databases. Patients with PAAD were divided into high- and low-risk categories according to the median risk score, with those in the high-risk group demonstrating a more unfavorable survival outcome in all three cohorts, with higher rates of gene mutations (e.g., KRAS, CDKN2A), increased levels of immunosuppressive cells (macrophages, Th2 cells, regulatory T cells), and heightened sensitivity to Acetalax and Selumetinlb. Conclusions: Abnormal metabolism of glycosphingolipids in epithelial cells may promote the development of PAAD. A model utilizing a gene signature associated with epithelial glycosphingolipids metabolism has been established, serving as a valuable indicator for the prognostic stratification of patients with PAAD.

## 1. Introduction

Pancreatic cancer is considered one of the most lethal types of cancer globally, showing a five-year survival rate of under 5% for all stages [1]. Chemotherapy remains the primary treatment for pancreatic cancer patients, but only a minority benefit from it as most develop rapid and common chemoresistance, leading to a poor prognosis [2]. Therefore, developing reliable prognostic biomarkers for patient stratification and treatment is crucial.

Recent evidence highlights that abnormal metabolism in cancer cells is not just a hallmark but also a root cause of tumors [3]. Malignant cells alter metabolic pathways to support their survival, uncontrolled growth, and division. Recent studies have shown that metabolic changes controlled by epigenetic histone acetylation can promote pancreatic tumorigenesis and metastasis, underscoring metabolism’s critical role in cancer development [4,5]. Moreover, altered metabolism in pancreatic cancer is closely associated with chemoresistance [6], radioresistance [7], and immunosuppression [8]. Different metabolic subgroups of pancreatic cancer have been identified based on glycolytic and cholesterogenic gene expression, correlating with survival and responses to chemotherapy [9,10]. Therefore, enhancing our understanding of pancreatic cancer’s metabolic characteristics could offer innovative and personalized therapeutic avenues.

Pancreatic cancer is defined by a robust desmoplastic response encompassing cancerous epithelial cells [11]. Research indicates that the interaction between the epithelial and stromal compartments promotes the aggressive nature of this disease [12]. The epithelial-to-mesenchymal transition (EMT) frequently serves a crucial function in pancreatic cancer progression and metastasis. The increased glucose uptake and lactate secretion seen under different EMT conditions suggest the occurrence of metabolic reprogramming during pancreatic cancer cell EMT [13]. It has been shown that the transition in metabolism from using oxidative phosphorylation to relying on glycolysis regulates the invasion-metastasis cascade in pancreatic cancer cells through boosting tumor angiogenesis, EMT, and metastatic growth in distant organs [14].

Studies employing single-cell RNA sequencing (scRNA-seq) on human tumors are valuable for uncovering tumor-related mechanisms, offering fresh insights into tumor diversity and unique subsets. In pancreatic cancer samples, both from humans and mice, scRNA-seq analysis have revealed up to seven unique transcriptomic groupings of malignant epithelial cell subsets, illustrating the diversity within such cells [15,16,17]. For this study, scRNA-seq data on pancreatic adenocarcinoma (PAAD) were sourced from publicly available Gene Expression Omnibus (GEO) databases. The research focused on the epithelial cell subclusters and metabolic activities. Subsequently, an epithelial cell-based risk signature was identified. Further analysis was conducted on clinical survival, immune infiltration, and drug responsiveness based on this signature. Finally, a new nomogram was created, which integrates the risk profile based on epithelial cells with clinicopathological characteristics, to assist in utilizing epithelial cell metabolic characteristics for PAAD prognosis in a clinical setting. These findings offer a promising prognostic and a new perspective for tailoring individual treatment plans for patients with PAAD.

## 2. Materials and Methods

### 2.1. Single-Cell Sequencing Data Quality Control and Processing

In this study, scRNA-seq data from GSE212966 were accessed from the GEO repositories. The raw data were then imported utilizing the Seurat package (version 4.2.0). Single cells were initially filtered, necessitating a minimum expression of each gene in three cells and a minimum expression of 200 genes in each cell. Cells expression genes ranging from 200 to 6000 were retained, with the proportion of mitochondria restricted to below 15%. Dimensionality reduction and cluster identification were carried out utilizing Uniform Manifold Approximation and Projection (UMAP) after conducting Principal Component Analysis (PCA), mapping cell populations on a two-dimensional plot. Subsequently, the “FindAllMarkers” function was utilized to detect differentially expressed genes (DEGs) within individual clusters. This process involved applying a filter with an absolute log2 fold change (FC) of 0.25 and a minimum cell population fraction of 0.25 within each group.

Cell types were annotated using the “CellMarker 2.0” tool for clustering analysis. Fisher’s exact test was conducted to identify significant cell groups in both tumor and normal tissues. Various functions such as FeaturePlot, DotPlot, and VlnPlot were employed to visualize the expression, distribution, and characteristic genes of tumor cells.

Functional enrichment analysis including Gene Ontology (GO) and Kyoto Encyclopedia of Genes and Genomes (KEGG) pathway analysis was conducted using the clusterProfiler R package (version 3.14.3). A significance level of less than 0.05 was utilized for determining statistical enrichment in biological processes (BPs), molecular functions (MFs), and cellular components (CCs).

### 2.2. Single-Cell Metabolic Analysis

The “scMetabolism” R package (v0.2.1) was applied with the VISION method for visualizing and quantifying the metabolic diversity of individual cells within each cluster. This package includes 85 KEGG pathways and 82 REACTOME pathways to facilitate a thorough analysis of metabolic activity among various NMF clusters [18]. The outcomes were depicted using the DotPlot.metabolism function.

### 2.3. Risk Model Construction

Univariate Cox regression analysis and Kaplan–Meier survival analysis were carried out to pinpoint DEGs that correlate with overall survival (OS). For this analysis, DEGs were identified via the survival R package (v3.3.1) with a significance level of less than 0.05. Following this, LASSO regression analysis was employed to determine the best prognostic genes for constructing a risk model. The ultimate risk model was established via multivariate Cox regression, utilizing the survival package with the chosen DEGs. The per-patient risk score was computed utilizing the formula: risk score = Ʃ(βi × Expi), where βi represents the LASSO coefficient of each gene and Expi denotes the expression value of each candidate gene. Using the median risk scores, patients diagnosed with PAAD were divided into high-risk and low-risk subgroups for an additional investigation.

### 2.4. Risk Model Evaluation and Validation

The risk score was utilized to assess the prognosis of patients with PAAD in the TCGA-PAAD training dataset, as well as in external validation sets ICGC-PAAD-US (*n* = 112) and GSE71729 (*n* = 114). A comparison between high-risk and low-risk individuals was conducted on the risk score curve, patient distribution, and selected gene expression levels for the prognostic model using the tinyarray R package (v2.2.9).

Survival curves at 1, 3, and 5 years were plotted, and the Area Under the Curve (AUC) was calculated using the survival (v3.3.1), survminer (v0.4.9), and timeROC (v0.4) R packages. Univariate and multivariate Cox analyses were carried out to investigate the independent prognostic value of the risk score, irrespective of clinical characteristics. The variables identified as statistically significant (*p* < 0.05) in the univariate Cox analysis underwent an additional evaluation in the multivariate Cox analysis. Variables that retained a significance level of *p* < 0.05 in the multivariate analysis were deemed as independent prognostic factors for PAAD.

### 2.5. Nomogram Model Construction

Nomogram is a visual tool that integrates various variables to forecast a specific outcome. In this study, a nomogram was created to assess the risk by utilizing the rms R package (v6.6-0). The characteristics considered in this evaluation were age, N, T, grade, and risk score. Furthermore, the clinical applicability of the nomogram was assessed using Decline Curve Analysis (DCA) with the ggDCA R package (v1.2).

### 2.6. Mutation Landscape Analysis

The mutation and clinical data of patients with TCGA were retrieved from The Pan-Cancer Atlas, which is available at https://gdc.cancer.gov/about-data/publications/pancanatlas (accessed on 18 January 2024). A waterfall plot was generated to visualize the mutation profiles of genes with the highest 5 mutation frequencies in patients with PAAD via the “maftools” R package (v2.14.0).

### 2.7. Immune Cell Infiltration Estimation and Immune Subtype Analysis

Firstly, the “ESTIMATE” algorithm with “limma” and “estimate” R packages was utilized to assess the TME scores, including immune infiltration, the stromal score, and the estimate score of each patient. In addition, the GSVA R tool, employing the ssGSEA technique, was utilized to quantify the abundance of the immune cell infiltration of each patient. Furthermore, the XCELL, QUANTISEQ, TIMER, and CIBERSORT algorithms were conducted to analyze the immune cell infiltration levels using the R packages immunedeconv (v2.1.3) and CIBERSORT (v0.1.0). The immune subtypes of individuals with TCGA-PAAD, which sorted tumors into six immune subgroups including C1 (Wound Healing), C2 (IFN-Dominant), C3 (Inflammatory), C4 (Lymphocyte Depleted), C5 (Immunologically Quiet), and C6 (TGF-Dominant) according to the previous research [19], were identified using the R package ImmuneSubtypeClassifier (v0.1.0).

### 2.8. Drug Response Prediction

Data regarding cell line expression and IC50 values were extracted from GDSC2, which is recognized as the most extensive pharmacogenomics database, encompassing information on 805 cell types and their reactions to 198 anti-tumor medications. The oncoPredict R tool was utilized to predict how the individuals with TCGA-PAAD would respond to these drugs. The relationship between drug sensitivity and risk score was computed with the R tool psych (v2.3.3) and visually represented using the R tools ggstatsplot and ggpubr.

### 2.9. Statistical Analysis

R software (v4.2.3) was employed for statistical analysis. Mean values across diverse groups were compared using Student’s *t*-test, while correlations were evaluated through Pearson correlation analysis. Kaplan–Meier survival analysis utilized the log-rank test, with statistical significance determined based on an adjusted *p*-value below 0.05.

## 3. Results

### 3.1. Analysis of Single-Cell Sequencing

**Figure 1:**
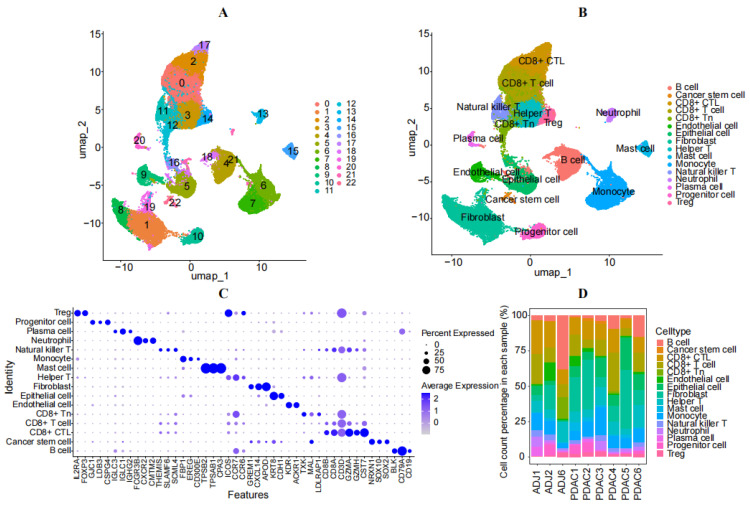
Identification of cell clusters using scRNA-seq data from patients with PAAD. (**A**) UMAP map displaying the distribution of 23 cell subgroups after clustering. (**B**) UMAP map showing the annotation results of the cell subgroups. (**C**) Dot plot depicting the expression profiles of marker genes in each cell type. (**D**) Cumulative histogram illustrating the distribution of cell types in patients with cancer compared to the control group.

After quality control, a sum of 43,580 cells were detected, leading to the identification of twenty-three unique clusters, following PCA and UMAP analysis, which was conducted utilizing a standard Seurat pipeline (Figure 1A). The clusters were annotated using previously reported canonical cell markers and the CellMarker database. Sixteen cell subgroups were characterized as B-cells, CD8+T-cells, CD8+CTLs, CD8+Tn, Helper T cells, natural killer T cells, epithelial cells, monocytes, neutrophils, fibroblasts, mast cells, plasma cells, Progenitor cells, Treg cells, endothelial cells, and cancer stem cells (Figure 1B). The DotPlot displays the expression and distribution of specific markers for each cell subgroup (Figure 1C). The percentage of each cell type in every sample was calculated and is listed in Table S1. Among them, epithelial cells and fibroblasts had the highest subpopulations, while CD8+CTLs had the lowest cell numbers in the tumor (Figure 1D). This suggests that increased epithelial cells and fibroblasts may play important roles in cancer invasion and metastasis.

**Figure 2:**
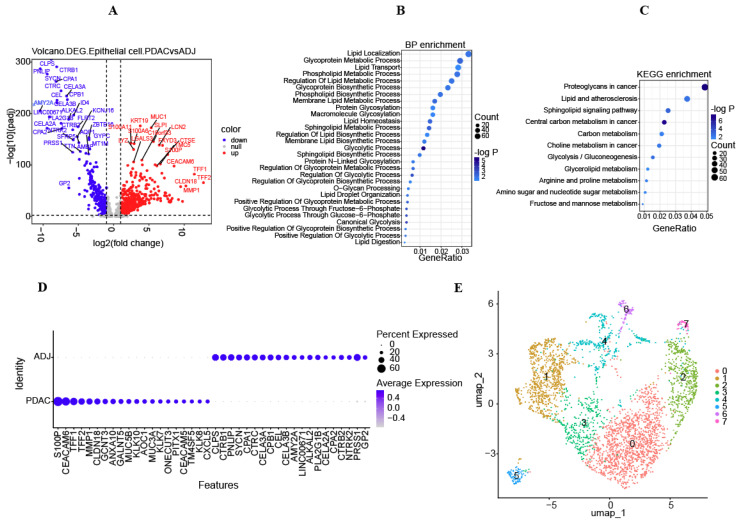
Differential expressed genes (DEGs) in epithelial cells based on scRNA-seq data. (**A**) Volcano plot presenting DEGs in epithelial cells from cancer and normal tissues. (**B**,**C**) Bubble plots showing the BP and KEGG functional enrichment pathways of DEGs. (**D**) Dot plot displaying the top 20 marker genes among the DEGs. (**E**) UMAP map demonstrating the distribution of eight epithelial cell clusters after clustering.

A total of 4834 DEGs were successfully achieved in epithelial cells from pancreatic cancer compared to normal paired tissue, with 2417 increased DEGs and 2417 decreased DEGs (Figure 2A and Table S2). GO enrichment analysis revealed that DEGs were primarily associated with the processes such as lipid localization, lipid transport, glycoprotein metabolic pathway, phospholipid metabolism, and lipid metabolism regulation (Figure 2B). KEGG enrichment analysis revealed that the enriched pathways for DEGs included proteoglycans in the cancer signaling pathway, lipid and atherosclerosis signaling pathway, sphingolipid signaling pathway, and glycolysis/gluconeogenesis (Figure 2C). Then, the epithelial cells underwent sub-clustering using Seurat. A visualization of the UMAP clusters revealed that the epithelial cells were categorized into eight subgroups (Figure 2E). The expression of crucial marker genes for epithelial cells was depicted using bubble plots (Figure 2D).

### 3.2. Metabolic Characteristics of Epithelial Cells and Screened DEGs

**Figure 3:**
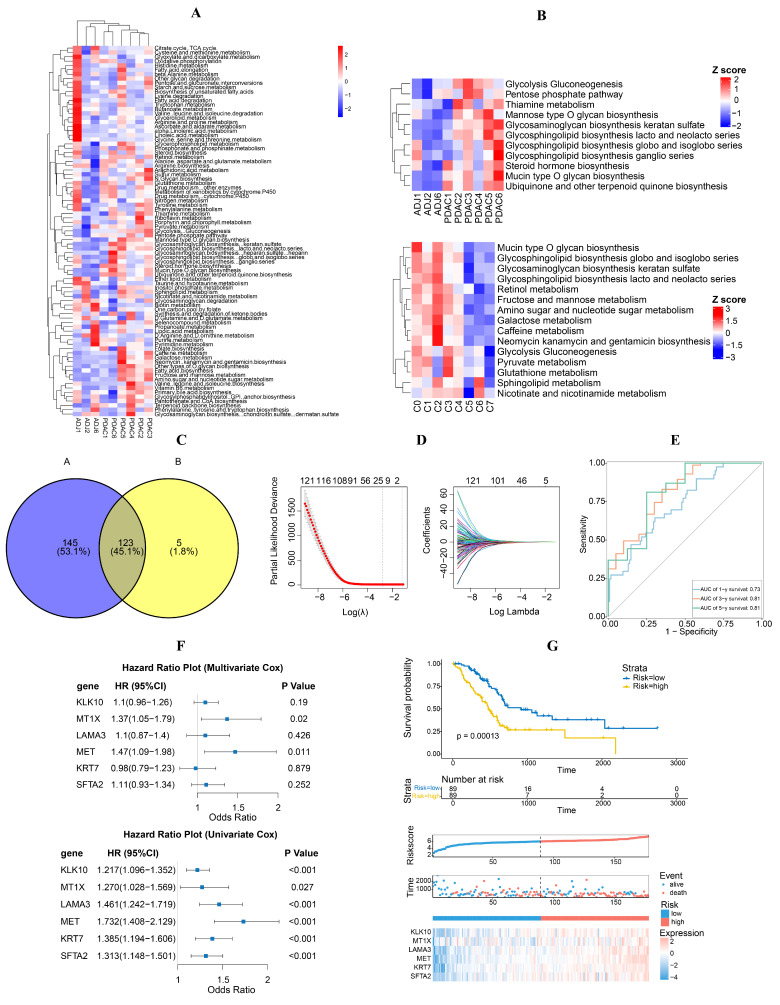
Risk model developed based on the glycosphingolipids metabolic enrichment in epithelial cells. (**A**) Heatmap showing metabolic functional enrichment analysis of cancer and normal tissues using the “ReactomeGSA” package (version 3.10). (**B**) Heatmap displaying glycosphingolipids and related metabolic enrichment in epithelial cells, which were clustered into eight subgroups labeled with C0–C7 via PCA and UMAP. (**C**) Venn plot identifying common DEGs between prognostic values in univariate Cox regression and Kaplan–Meier survival analysis. A indicated the DEGs from prognostic values in univariate Cox regression, B indicated the DEGs from Kaplan–Meier survival analysis. (**D**) Lasso regression profiles to prevent overfitting and 10-fold cross-validation of variable selection with Lasso. (**E**) Time-dependent ROC curve analysis in the TCGA cohort. (**F**) Forest plot of univariate and multivariate Cox regression analyses of the DEGs. (**G**) Distribution of risk scores and patient survival between low- and high-risk groups in the TCGA cohort.

To explore the metabolic profile of epithelial cells in pancreatic cancer, the “scMetabolism” package was employed to calculate the scores of active metabolic pathways. A noticeable metabolic shift was observed from normal to cancerous states (Figure 3A). In pancreatic cancer epithelial clusters, elevated levels were detected in glycolysis, fructose and mannose metabolism, glycosaminoglycan biosynthesis, mucin type O-glycan biosynthesis, ubiquinone, and other terpenoid quinone biosynthesis, as well as glycosphingolipid biosynthesis. Conversely, taurine, hypotaurine, and biotin metabolism were lower in the cancer epithelial cluster (Figure 3B). Moreover, glycosphingolipid biosynthesis was heightened in most of the pancreatic cancer epithelial clusters (Figure 3B).

Subsequently, to identify DEGs associated with glycosphingolipid metabolism in epithelial cells, we intersected the DEGs in epithelial cells between cancerous and normal tissues with the DEGs in the epithelial cells between those displaying high glycosphingolipid metabolism and those displaying low glycosphingolipid metabolism (Table S3). This analysis resulted in the selection of 425 DEGs related to glycosphingolipid metabolism for further investigation (Table S4).

### 3.3. Development, Assessment, and Verification of Risk Models

The potential prognostic value of DEGs was determined through univariate Cox regression analysis. In total, 268 DEGs with a prognostic value were discovered through univariate Cox regression analysis (Table S5). Additionally, 128 DEGs with a prognostic value were discovered with Kaplan–Meier survival analysis (Table S6). Subsequently, 123 DEGs that overlapped were selected for further analysis using LASSO regression to identify the optimal candidates for Cox’s proportional hazards model (Figure 3C,D). This process resulted in the selection of six genes (KLK10, MT1X, LAMA3, MET, KRT7, SFTA2) to construct the final multigene risk model.

The association between the risk genes and the OS of individuals with PAAD was affirmed through both univariate and multivariate Cox regression analyses (Figure 3F). A particular formula was utilized to calculate the risk score based on the gene coefficients. Risk score = 0.09194086 × KLK10 + 0.31770488 × MT1X + 0.09734123 × LAMA3 + 0.38594268 × MET − 0.01714449 × KRT7 + 0.10732803 × SFTA2. Following the computation of the risk score for every individual in the TCGA-PAAD training database, individuals were classified into high- and low-risk groups based on the median risk score. Gene expression analysis showed that the genes were upregulated in the high-risk group (Figure 3G).

**Figure 4:**
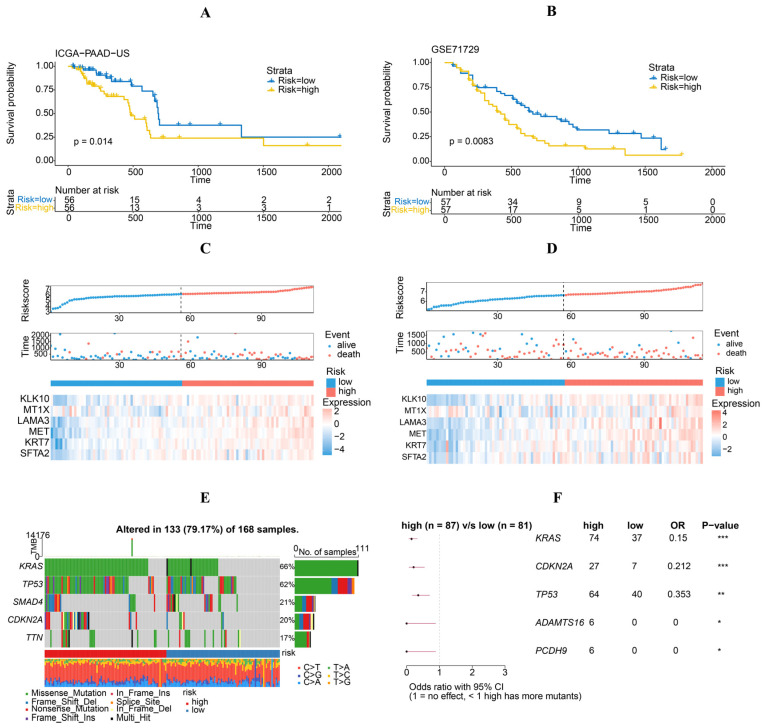
External validation of the risk model in PAAD. (**A**,**B**) Kaplan–Meier curves of high- and low-risk patients. (**C**,**D**) Risk score and patient distribution, as well as the expression of risk genes in ICGC-PAAD-US and GSE71729 cohorts. (**E**) Variation landscape of the top five mutated genes in the high- and low-risk groups. (**F**) Ratios of five gene mutations in the high- and low-risk groups. “*” mean *p* < 0.05, “**” mean *p* < 0.01, “***” mean *p* < 0.001.

Kaplan–Meier survival analysis revealed that individuals in the high-risk category experienced a notably worse prognosis in comparison to those in the low-risk group (Figure 3G). Furthermore, the receiver operating characteristic (ROC) curve analysis indicated that the risk model demonstrated high accuracy and specificity in its predictive capabilities of OS in patients with PAAD, with 1-, 3-, and 5-year area under AUC values of 0.73, 0.81, and 0.81, respectively (Figure 3E). We also confirmed the prognostic capability of the risk model using two external datasets, ICGC-PAAD-US and GSE71729. The high-risk group exhibited a significantly poorer prognosis, aligning with the findings observed in the training dataset. Furthermore, the gene expression profile of the risk genes closely resembled that of the TCGA cohort (Figure 4A–D). The consistent outcomes observed across different cohorts indicate that the gene signature could potentially function as a standalone predictive marker for patients with PAAD.

**Figure 5:**
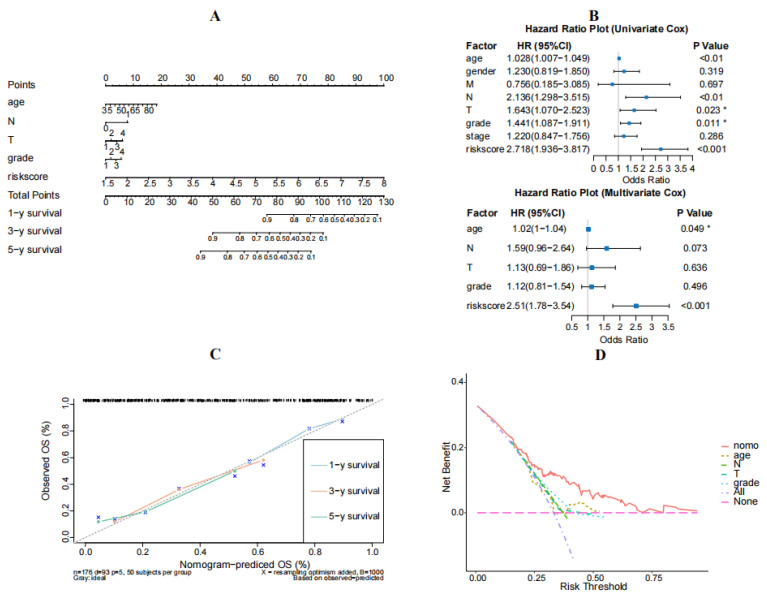
Development of a nomogram for predicting the prognosis of HCC. (**A**) Nomogram model integrating risk score and stage. (**B**) Univariate and multivariate Cox analyses of risk score and clinicopathological characteristics. “*” mean *p* < 0.05 (**C**) Calibration curves for nomogram-predicted 1-, 3-, and 5-years overall survival. (**D**) Decision curve nomogram.

A predictive nomogram was created to forecast the 1-, 3-, and 5-year survival rates among the patients with TCGA-PAAD, based on key prognostic factors like age, age, N-stage, T-stage, grade, and risk score (Figure 5A). Additionally, both univariate and multivariate Cox regression analyses validated a strong association between the risk score and OS for patients with PAAD (Figure 5B). This indicates that the risk score has the potential to be employed as a standalone prognostic factor. Within the nomogram, the risk score model held the highest level of significance compared to the other factors mentioned. The calibration curves confirmed that the nomogram’s performance in patients with TCGA-PAAD was similar to an ideal model (Figure 5C). Additionally, the DCA curves illustrated the nomogram’s superior predictive power over clinical factors for 1-, 3-, and 5-year overall survival (Figure 5D).

### 3.4. Mutation Profile and Drug Sensitivity Variances between High-Risk and Low-Risk Cohorts

This research aimed to ascertain variances in cancer-associated gene mutations between high-risk and low-risk cohorts. Gene mutations were tallied in each group, revealing KRAS, TP53, SMAD4, CDKN2A, and TTN as the top five mutated genes in both groups. The mutation rates of KRAS, CDKN2A, TP53, ADAMTS16, and PCDH9 were notably greater in high-risk individuals than in low-risk individuals (Figure 5E,F). These findings suggest that specific gene mutations can distinguish patients with PAAD based on their molecular profiles and mutation patterns.

**Figure 6:**
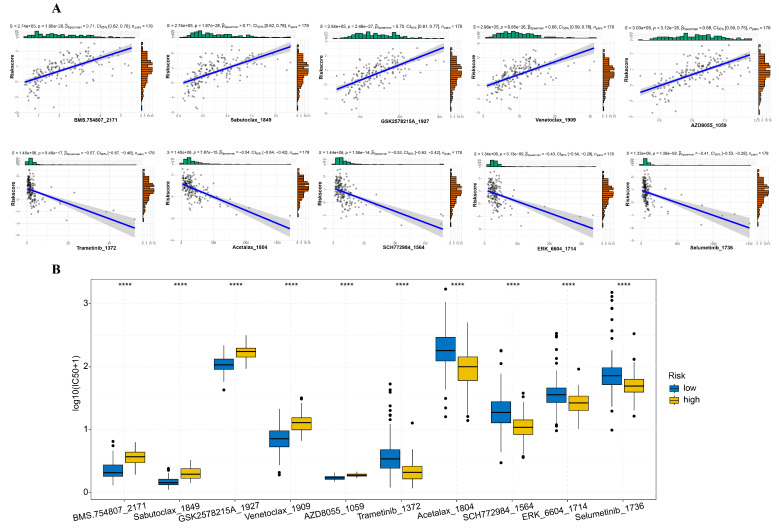
Correlation between drug sensitivity and risk score. (**A**) Spearman’s correlation analysis between drug IC50 and risk score. (**B**) Examination of differences in drug IC50 between high- and low-risk groups using the Wilcoxon test and presentation through a box plot. “****” mean *p* < 0.0001.

To evaluate the drug response potential between the two risk groups, we measured the IC50 values of 198 chemotherapy drugs or inhibitors. Through Spearman’s correlation analysis, we found positive correlations between BMS.754807, Sabutoclax, GSK2578215A, Venetoclax, and AZD8055 with the risk scores. Conversely, negative correlations were observed with Trametinib, Acetalax, SCH772984, ERK_6604, and Selumetinib (Figure 6A). Additionally, the drugs positively correlated were associated with markedly lower IC50 levels in the low-risk group in comparison to the high-risk group (Figure 6B).

### 3.5. Immune Characteristics of High- and Low-Risk Groups

**Figure 7:**
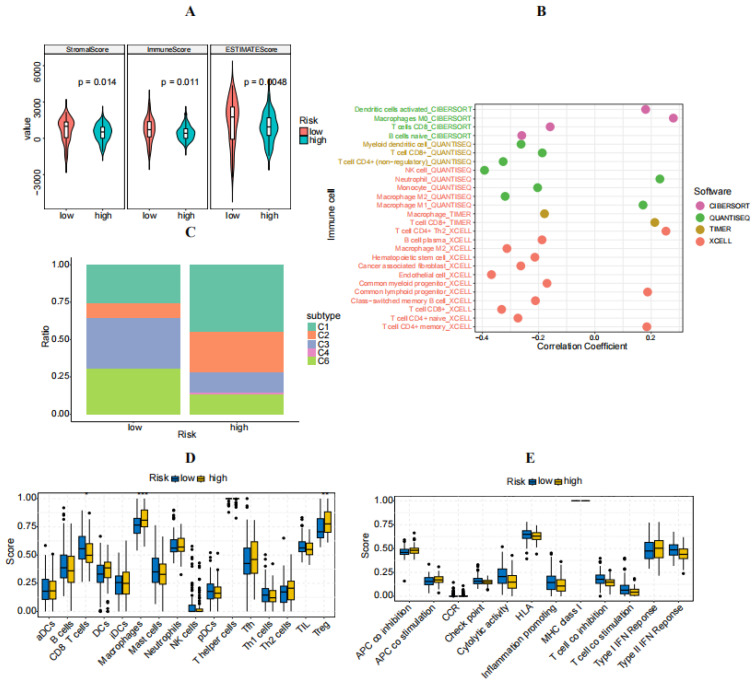
Immune characteristics of high- and low-risk groups. (**A**) Comparison of stromal score, immune score, and estimate score between high- and low-risk groups. (**B**) Correlation between immune cell abundance and risk score. (**C**) Distribution of patients with PAAD in different immune subtypes. (**D**) Comparison of immune cell infiltration between high- and low-risk groups. (**E**) Immune function scores between high- and low-risk groups.

In this section, the immune characteristics of individuals in high- and low-risk categories were investigated. Initially, a comparison was made of the immune cell infiltration levels in both the high- and low-risk groups, demonstrating an increased stromal score, immune score, and estimate score in the low-risk group (Figure 7A). A further examination using the CIBERSORT algorithm revealed an increased presence of B cells, CD8 T cells, Th1 cells, and TIL cells in low-risk patients. Conversely, high-risk individuals showed elevated levels of DCs, macrophages, neutrophils, Tfh cells, Th2 cells, and regulatory T cells (Treg) (Figure 7D). High-risk patients also displayed reduced cytolytic activity, HLA expression, T cell co-inhibition, and type II IFN responses, suggesting an immunosuppressive tumor microenvironment (Figure 7E). Additional analysis identified a negative relationship between risk score and B cells and macrophage M2 cells (Figure 7B). A subsequent categorization of patients with PAAD into C1-C6 immune subtypes highlighted that the C4 (lymphocyte-depleted) subtype was unique to the high-risk group, showing a Th1-inhibited pattern and enhanced M2 reaction, suggesting an immunosuppressed state. The nonexistence of the C5 (immunologically quiet) subtype in both sets of patients indicated a malignancy feature of PAAD (Figure 7C).

### 3.6. Validation of Gene Expression in PAAD Tissues

**Figure 8:**
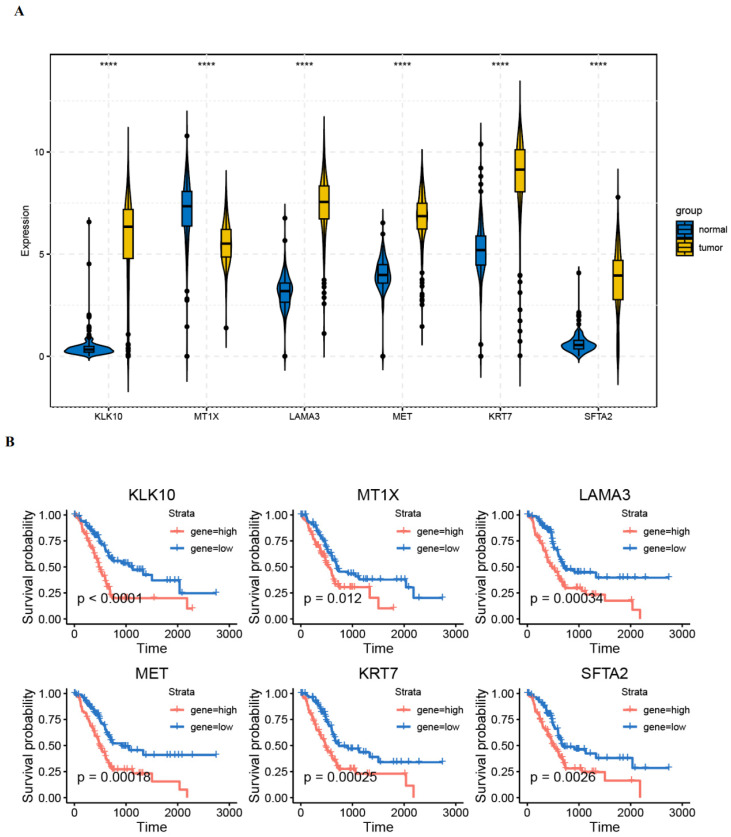
Expression validation of risk genes in PAAD tissues. (**A**) mRNA levels of risk genes in TCGA-GTEx cohort. “****” mean *p* < 0.0001. (**B**) Kaplan–Meier curve of high- and low-risk patients based on the expression of risk genes in the TCGA cohort.

In our continued investigation, we delved into the expression patterns of risk genes in PAAD tissues. An examination of mRNA levels within the TCGA-GTEx cohort demonstrated a notable increase in KLK10, LAMA3, MET, KRT7, and SFTA2, alongside a decrease in MT1X in tumor samples compared to their normal counterparts (as illustrated in Figure 8A). Furthermore, Kaplan–Meier analysis—which utilized the expression profiles of these high-risk genes within the TCGA dataset—revealed a consistent negative correlation, connecting the expression levels of all genes with the OS of patients with PAAD (Figure 8B).

## 4. Discussion

In this study, we investigated the diversity of the tumor microenvironment (TME) in pancreatic cancer, primarily focusing on the characterization and classification of epithelial cells using single-cell RNA sequencing (scRNA-seq) data. Moreover, we developed a prognostic model for PAAD based on epithelial glycosphingolipid metabolic data from scRNA-seq and public datasets.

Glycosphingolipids (GSLs), also known as glycolipids, are carbohydrate chains linked to lipid molecules. Together with sphingolipids, sterols, and proteins, they constitute plasma membrane lipid rafts essential for maintaining membrane integrity and creating specific recognition sites [20]. Abnormal GSL expression is a unique characteristic of cancer and stromal cells within tumor microenvironments [21]. A member of the glycolipid transfer protein family, Ceramide-1-phosphate transfer protein (CPTP), is involved in the regulation of both autophagy and inflammation and has been linked to promoting growth and metastasis in pancreatic cancer cells through sphingolipid metabolite ceramide and the PI4K/AKT signaling pathway [22]. The epithelial–mesenchymal transition (EMT) is another crucial process involved in the metastatic advancement of primary tumors [23,24]. GSLs also participate in the EMT process, with ganglioside GM3 affecting the regulation of TGF-1-induced EMT in human lens epithelial cells [25,26].

The outcomes of GO enrichment and KEGG enrichment analysis suggested that DEGs were mainly enriched in the glycoprotein metabolic process, sphingolipid signaling pathway, and glycolysis/gluconeogenesis. Moreover, we utilized scMetabolism to visualize and quantify the metabolic diversity of individual epithelial cells. The expression levels of pathways such as glycolysis, fructose and mannose metabolism, mucin type O-glycan biosynthesis, glycosaminoglycan biosynthesis, ubiquinone and other terpenoid quinone biosynthesis, and glycosphingolipid biosynthesis were higher in the cancer epithelial clusters compared to the normal ones. These findings suggest that glycosphingolipids metabolic alterations in the epithelial cells of PAAD may be associated with tumorigenesis and metastasis.

We utilized bioinformatic methods to develop a gene signature in PAAD using TCGA data. This signature was evaluated for its efficacy in predicting survival, immune activity, mutation status, and response to chemotherapy. The final risk model encompassed six genes: KLK10, MT1X, LAMA3, MET, KRT7, and SFTA2. KLK10 showed significantly elevated levels in PAAD tissues, particularly in patients with lymphatic and distant metastasis. Abnormal expression of KLK10 was strongly associated with a poorer prognosis and shorter survival [27]. MT1X, a gene involved in the lysosomal pathway, exhibited a lower expression in esophageal cancer cells when compared to normal esophageal epithelial cells. Knocking down MT1X significantly enhanced the growth rate of esophageal cancer cells [28]. The upregulation of LAMA3 influenced the growth, attachment, movement, and transition from epithelial to mesenchymal states in cholangiocarcinoma cells [29]. MET, a critical tyrosine kinase, played a vital role in the initiation and progression to drive cancer development by enhancing cell proliferation, morphogenesis, and survival. It was overexpressed and contributed significantly to the progression of PAAD [30,31]. KRT7 displayed atypical expression in different types of cancers like esophageal squamous cell carcinoma and colorectal cancer. Additionally, its involvement in the invasion and metastasis of cervical cancer contributed to the malignant advancement of the disease [32,33]. SFTA2 was identified as a prognostic gene closely associated with the pathological stages of PAAD in an integrated transcriptome meta-analysis [34].

After each patient’s risk score was computed, the median risk score was utilized to divide patients into high- and low-risk categories. Kaplan–Meier survival analysis demonstrated a significantly worse prognosis for patients classified in the high-risk group in contrast to those in the low-risk group. Furthermore, the ROC curve indicated AUC values of 0.73, 0.81, and 0.81 for 1-, 3-, and 5-year OS, respectively, suggesting that the risk model outperformed previous reports [35,36]. Additionally, the model was validated as a robust prognostic biomarker for PAAD across three independent cohorts. To further elucidate the impact of the risk model on patient survival, a nomogram was established, highlighting the prominence of the risk score model among variables such as age, N stage, T stage, grade, and risk score. These findings collectively support the development of an outstanding risk model derived from a gene signature associated with epithelial glycosphingolipid metabolism for stratifying the risks of patients with PAAD.

In this study, gene mutations, drug sensitivity, and immune infiltration features were contrasted between the high-risk and low-risk cohorts. Consistent with previous research, TP53 gene mutations were notably more prevalent in the high-risk group [37]. Additionally, differences in drug sensitivity potential were observed between the two groups. Immune infiltration analysis revealed higher levels of the presence of CD8 T cells, B cells, TIL cells, and Th1 cells in low-risk patients, whereas increased levels of DCs, Tfh cells, neutrophils, macrophages, Th2 cells, and regulatory T cells (Tregs) were noted in high-risk patients, indicating an immunosuppressive tumor microenvironment in the high-risk group. These results indicate that the risk model may function as a dependable biomarker for predicting the molecular characteristics, immune status, and chemotherapy sensitivity of patients with PAAD.

As far as we know, we are the first ones that focused on the epithelial cell subclusters and metabolic activities in PAAD as well as being the first to have developed a new prognostic risk score model to effectively predict the prognosis, molecular characteristics, immune status, and sensitivity to chemotherapy drugs in patients with PAAD. However, it is important to recognize certain limitations in this study. First and foremost, there is a deficiency in experimental validation and external validation in clinical cohorts. Secondly, this study’s outcomes heavily relied on data quality from publicly available databases.

In conclusion, the aberrant metabolism of glycosphingolipids in epithelial cells may contribute to the development of PAAD. We have developed a model based on a gene signature associated with epithelial glycosphingolipid metabolism, which may serve as a valuable tool for the prognostic stratification of patients with PAAD.

## Data Availability

The original contributions presented in the study are included in the article/Supplementary Materials, further inquiries can be directed to the corresponding author.

## Supplementary Materials

The following supporting information can be downloaded at: https://www.mdpi.com/article/10.3390/diagnostics14111094/s1, Table S1. Percentage of the cell types in the two groups; Table S2. DEG Epithelial cell PDAC vs. ADJ; Table S3. DEG epicell Metabolism high vs. low; Table S4. DEGs intersected from epicell PDAC vs. ADJ and Metabolism high vs. low; Table S5. DEGs from prognostic values in univariate Cox regression; Table S6. DEGs from Kaplan–Meier survival analysis.
